# Anaplastic astrocytoma mimicking progressive multifocal leucoencephalopathy: a case report and review of the overlapping syndromes

**DOI:** 10.1186/s12885-017-3415-1

**Published:** 2017-06-19

**Authors:** Ema Kantorová, Michal Bittšanský, Štefan Sivák, Eva Baranovičová, Petra Hnilicová, Vladimír Nosáľ, Daniel Čierny, Kamil Zeleňák, Wolfgang Brück, Egon Kurča

**Affiliations:** 1 Clinic of Neurology, Jessenius Faculty of Medicine, Comenius University in Bratislava, Kollárova 2, 03659 Martin, Slovak Republic; 2 Department of Medical Biochemistry, Jessenius Faculty of Medicine, Comenius University in Bratislava, Kollárova 2, 03659 Martin, Slovak Republic; 3 Clinic of Radiodiagnostics, Jessenius Faculty of Medicine, Comenius University in Bratislava, Kollárova 2, 03659 Martin, Slovak Republic; 4 Institut für Neuropathologie Universitätsmedizin Göttingen, Robert-Koch-Str, 40 37075 Göttingen, Germany

**Keywords:** Anaplastic astrocytoma, Multiple sclerosis, Tumefactive lesion, Progressive multifocal leukoencephalopathy, Immune reconstitution syndrome

## Abstract

**Background:**

Co-occurrence of multiple sclerosis (MS) and glial tumours (GT) is uncommon although occasionally reported in medical literature. Interpreting the overlapping radiologic and clinical characteristics of glial tumours, MS lesions, and progressive multifocal leukoencephalopathy (PML) can be a significant diagnostic challenge.

**Case presentation:**

We report a case of anaplastic astrocytoma mimicking PML in a 27-year-old patient with a 15-year history of MS. She was treated with interferon, natalizumab and finally fingolimod due to active MS. Follow-up MRI, blood and cerebrospinal fluid examinations, and biopsy were conducted, but only the latter was able to reveal the cause of progressive worsening of patient’s disease.

**Conclusions:**

Anaplastic astrocytoma misdiagnosed as PML has not yet been described. We suppose that the astrocytoma could have evolved from a low grade glioma to anaplastic astrocytoma over time, as the tumour developed adjacent to typical MS plaques. The role of the immunomodulatory treatment as well as other immunological factors in the malignant transformation can only be hypothesised. We discuss clinical, laboratory and diagnostic aspects of a malignant GT, MS lesions and PML. The diagnosis of malignant GT must be kept in mind when an atypical lesion develops in a patient with MS.

## Background

Multiple sclerosis (MS) is a disabling inflammatory demyelinating disease of the central nervous system (CNS). Early initiation of immunotherapy and its adjustment in view of ongoing inflammatory disease activity is desirable [1]. The main treatment goals aim at terminating inflammation and at reducing axonal damage [1]. Establishing MS diagnosis and decisions about the initial and ongoing treatments should not be made until other disorders that could better explain neurological symptoms and signs are excluded [1]. Although glial brain tumors (GT) have occasionally been reported in patients with MS, with only about 80 cases described in medical literature so far [2–5], this co-occurrence is uncommon since MS is caused by putative CNS autoimmune mechanisms whereas brain neoplasms may depended on a subclinical immunosuppressive state [6]. However, the last 15 years have seen increased use of immunomodulatory therapies (IMT) for relapsing MS, and considerable progress in the development of new, much stronger IMT for MS [7]. That rises questions about long-term safety of IMT as well as their risks and benefits [7]. Currently we know that several IMT make patients more susceptible to developing dangerous brain infection caused by John Cunningham virus (JCV) called progressive multifocal leukoencephalopathy (PML) [8–12]. However, MRI findings of tumefactive demyelinating lesions (TDL), PML and GT can overlap [12–19]. The first appearance of atypical brain lesion in an MS patient should lead to more extensive investigation in order to exclude another disease [15–19]. However, in some cases only biopsy is capable to reveal the cause of atypical MRI lesion [20, 21].

Here we describe the case of a malignant GT in a patient with early onset of MS. To the best of our knowledge, anaplastic astrocytoma misdiagnosed as PML has not yet been described. We discuss diagnostic tools that can help in differential diagnosis.

## Case presentation

**Fig. 1:**
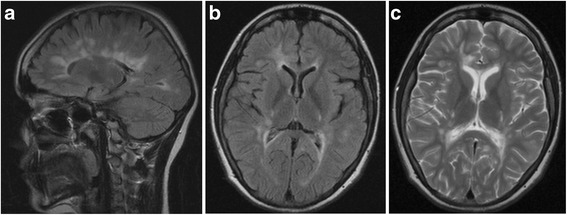
**a** FLAIR, Fluid-Attenuated Inversion Recovery, sagittal (2006): Periventricular high–signal intensity lesions exhibiting distribution of ovoid demyelinated periventricular lesions radially oriented to ventricles, which is typical for multiple sclerosis. **b** FLAIR, Fluid-Attenuated Inversion Recovery, transverse (2006): Atypical tumefactive periventricular demyelinated lesion connected to the frontal pole of the lateral ventricle. **c** T2w, T2-weighted MRI, transeverse (2006): Atypical hyperintensive tumefactive periventricular demyelinated lesion connected to the frontal pole of the lateral ventricle

**Fig. 2:**
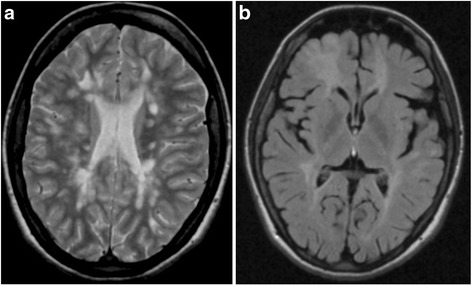
**a** Dual Fast SE, Dual Fast Spin-Echo, transverse (2011): Enlargement of the atypical hyperintensive tumefactive demyelinated lesion in the right frontal lobe. **b** FLAIR, Fluid -Attenuated Inversion Recovery, transverse (2011): Enlargement of the atypical tumefactive demyelinated lesion in the right frontal lobe

**Fig. 3:**
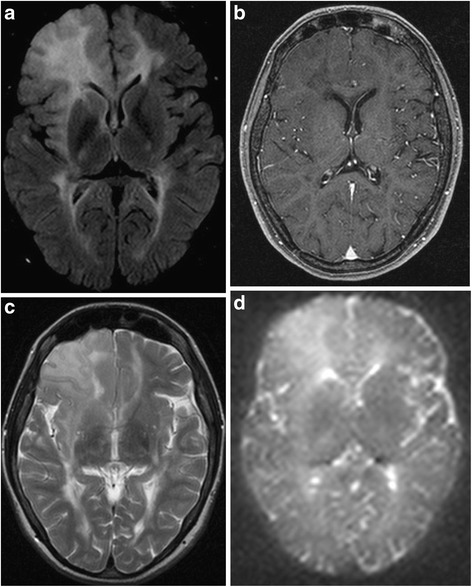
**a** FLAIR, Fluid-Attenuated Inversion Recovery, transverse (2013): Progression of the non-homogeneously hyper-intensive demyelinated lesion of the right frontal lobe, involving U-fibers. The lesion is well-defined to cortex, confluent with white mater, and irregular in shape. **b** T1w, T1-weighted MRI, transverse (2013): Hypointense irregular lesion at the rim of the right corner of the lateral ventricle in the right frontal lobe and several slightly hypointensive areas subcortically with no post-Gad enhancement. **c** T2w, T2-weighted MRI, transverse (2013): Irregular signal intensity within the lesion in the right frontal lobe. **d** DWI, Diffusion Weighted Imaging (2013): High signal intensity in the right frontal cortico-subcortical region and slightly increased signal in periventricular regions of both hemispheres

**Fig. 4:**
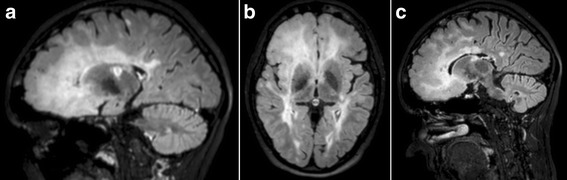
**a** FLAIR, Fluid-Attenuated Inversion Recovery, sagittal (2014): Large non-homogenous hyperintense lesion of the right frontal lobe involving demyelination and oedema with mild mass-effect. It is relatively sharply defined to grey matter and confluent with white matter. Glial tumour is undetectable. **b** FLAIR, Fluid -Attenuated Inversion Recovery, transverse (2014): Diffuse hyperintense lesion of the right frontal lobe - demyelination. It has mild mass-effect. It is well-defined to cortex and to white matter and irregular in shape. **c** 3D DIR, 3D Double Inversion Recovery, sagittal (2014): Multifocal cortical involvement in the right frontal cortex adjacent to demyelinated lesions, diffuse confluent hyperintensive lesion in cortico-subcortical fronto-polar region

**Fig. 5:**
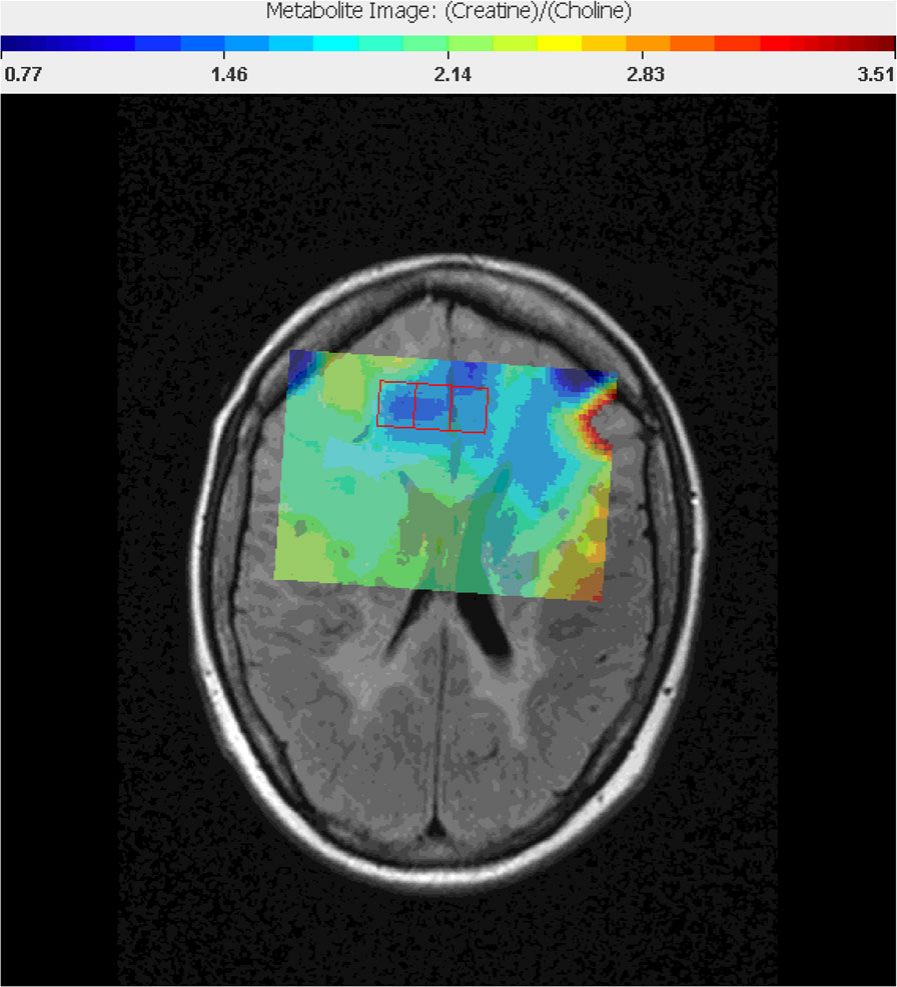
^1^HMRS, ^1^H–magnetic resonance spectroscopy (2015): 1H–MRS of the right and left frontal lobes - Creatine to Cholin maps, the decreased ratio may indicate tumorous tissue (red-contoured squares)

**Fig. 6:**
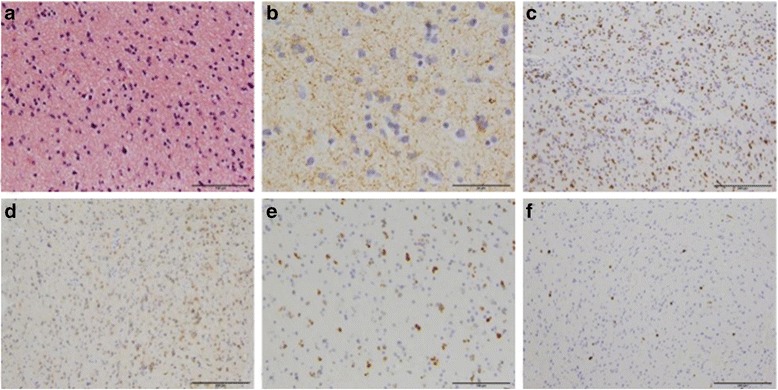
Histopathology findings. (HE, LFB-PAS, Bielschowsky, CD3, SV40, Olig2, GFAP, Vimentin, Nogo-A, Ki67, IDH1, p53): **a** The hematoxylin-eosin (HE) staining revealed grey and white matter with a markedly increased cellularity. Cells appeared pleomorphic and demonstrated a diffuse invasion into the CNS tissue. The tumour cells were embedded into a glial matrix. The nuclei showed a pronounced variation with respect to size and shape and depicted an increased nucleolar prominence. Mitosis was detectable. Signs of necrosis or microvascular proliferation were absent. **b** The immunohistochemical staining for Glial Fibrillary Acidic Protein (GFAP). Vimentin marked the majority of the tumour cells. **c** Some of the tumour cells were positive for Olig2. The tumour cells were not positive for NogoA. The proliferation ranged between 2 and 3% as determined by Ki67 immunohistochemistry. **d** The tumour cells were positive for isocitrate dehydrogenase1 (IDH1) and p53. **e, f** T-lymphocytes were not increased in the CD3 immunohistochemistry No SV40 positive cells were detected

**Fig. 7:**
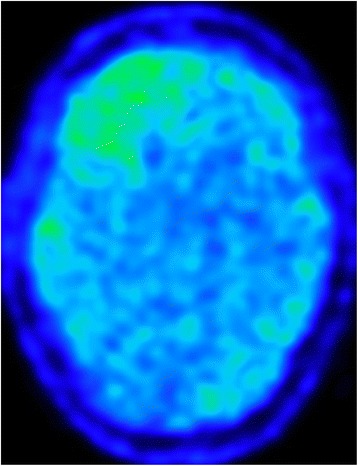
^11^-MET PET, ^11^Methyl-Methionine Positron Emission Tomography (2015): Increased uptake of ^11^C–methionine in anaplastic astrocytoma in the right frontal cortico-subcortical region, showing high proliferation index of the tumour. Lower uptake was also detected in left frontal and occcipital cortical areas

Here we report on a 27-year-old woman with the first neurological symptoms suggesting MS in 1999 at the age of 12. Her medical and psychosocial history was negative, but her family history was positive. Patient’s father is also treated for MS. The patient’s clinical timeline follows (Figs. 1, 2, 3, 4, 5, 6 and 7):

**Table:** 

1999	A 12-year-old girl with negative medical history presents with vestibular syndrome lasting three weeks
1999–2006	After having experienced several relapses with various symptoms (paresthesias of her left and right upper limbs, paresthesias of her distal limbs, weakness of upper limbs), she fulfilled McDonald criteria for definite relapsing-remitting MS due to demyelinating lesions in MRI (Fig. 1a, b, c), positive oligoclonal bands type 2 in cerebrospinal fluid (CSF) and positive visual evoked potentials (VEP).
2006 October	Several rounds of intravenous boluses of methylprednisolon were effective and the patient improved. When she started treatment by interferon beta Ia (Rebif® Merck-Serono), her Expanded Disability Status Scale (EDSS) was 2.0.
2008–2009	The treatment was interrupted due to her pregnancy in February 2008. In January 2009 she gave birth. During the early postpartum period the patient’s neurological status was unstable.
2009 January to April	The patient suffered three relapses (sensitive symptoms, left-sided hemiparesis, paraparesis of distal limbs) and her EDSS increased to 4.0 although she obtained several rounds of intravenous methylprednisolon.
2009 May	She resumed interferon beta Ia (Rebif® Merck-Serono) and reached remission.
2011 March to May	Her disease progressed again, her EDSS increased to 4.5 and follow-up MRI reflected clinical activity (Fig. 2a, b). Central quadruparesis was more pronounced in her left limbs and spinal ataxia varied over time between a need of assistance when walking longer distances and mild deficit. She responded well to intravenous methylprednisolon rounds.
2012 August	To stop the disease progression, she was indicated to natalizumab (Tysabri® Biogen Idec), receiving 12 infusions (august 2012 - september 2013).
2013 September	The follow-up brain 3.0 Tesla MRI showed enlargement of the lesion in the right frontal lobe, evaluated by radiologists as PML (Fig. 3a, b, c, d). We also noticed seroconversion of JCV antibodies, but JCV index was low (0.38) and CSF PCR of DNA revealed no copies of JCV (Focus diagnostics, CYPRESS, California, USA).
2013 December	Not meeting Slovak indication criteria, the patient ceased taking natalizumab. The patient started treatment with fingolimod (Gilenya® Novartis Pharmaceuticals UK). At that time she was quadruparetic, more prominent on the left side. She needed assistance due to wide-based gait and she had intermittent headaches (mild to moderate congestive-dull or pulsating headache located in bi-temporal areas, partially alleviated by analgesics) EDSS was 5.0.
2014 February	A follow-up 3.0 Tesla MRI of the brain showed enlargement of the prior frontal lobe lesion (Fig. 4a, b, c), misinterpreted as PML again. A new CSF examination showed normal proteinorhachia (0.22g/l) and cellularity (1lymphocyte), not increased lactate (1.91 mmol/l), positive oligoclonal bands type 2 (14 bands), and increased IgG index 1.44. The PCR test of DNA JCV was negative again (0 copies UNILABS, Denmark). We decided to continue with fingolimod.
2014 December	Over the following several months she developed new clinical symptoms: headache, sporadic epileptic seizures, disorientation. Immunomodulatory treatment was stopped. Repeated MRI was comparable with the MRI from February 2014, the atypical lesion in the right frontal lobe was in mild progression.
2015 May	^1^H-magnetic resonance spectroscopy (^1^H-MRS) detected decreased creatin to cholin ratio in several small areas of the frontal lobe, possibly suggesting tumorous mass (Fig. 5) Brain biopsy of the tumefactive lesion from the right frontal lobe. Histopathological findings revealed presence of anaplastic astrocytoma (Fig. 6a, b, c, d, e)
2015 June	The patient needed anti-oedematous (dexamethason or methylprednisolon, boluses of manitol) and anti-epileptic therapy (valproic acid and levetiracetam) due to repeated secondary generalized epileptic seizures and intracranial hypertension syndrome.
2015 August	Before starting oncological treatment, 11methyl-methionine positron emission tomography (^11^C MET PET) showed cortical localization of the brain tumor (Fig. 7). Patient’s condition remained unstable due to frequent epileptic seizures. Three weeks later she suddenly died during status epilepticus (23/Aug/2015).

## Discussion and conclusions

Our case shows that MS can have variable presentation therefore concurrence of MS with brain tumours may remain undetected for some time. One possible explanation is that an association of MS with overall risk of cancer has not been proved [22, 23]. One meta-analysis even identified a small but significant reductions of total cancer risk in patients with MS (odds ratio [OR] 0.92; 95% CI 0.87–0.97, *p* = 0.004) [24]. Another meta-analysis suggested lower overall co-occurrence of cancer in patients with MS [24]. On the other hand, a recent large systematic study evaluating risk and survival of brain tumours among patients with autoimmune disorders found higher standardised incidence of brain tumours in MS (OR 2.14; 95% CI 1.65–2,73) than in other autoimmunity disorders of the CNS [25]. The most frequent brain tumours were gliomas with OR 1.49; 95% CI 0.91–2.31. In this study, the risk of glioma-associated deaths in MS patients was relatively high (OR 2.3; 95% CI 1.47–3.61). In addition, the data on survival showed the same decrease for both benign and malignant types of tumours co-occurring with MS [25]. Curiously, MS was the only autoimmune disease for which the overall brain cancer and especially glioma-specific survival appeared to decrease in recent years [25]. One explanation could be that comorbidity weakens patient’s physical condition. Moreover in MS, glial tumours interfere with mortality burden due to reduced treatment options and lower capacity of the affected brain to resist tumour growth. Another explanation could be that brain tumours remain hidden in demyelinated brain tissue and therefore they are diagnosed later, in advanced stages, which was also our case.

At the beginning of our patient’s disease the pathological lesion was connected to the frontal pole of lateral ventricle stretching to the periphery dispersing into the white matter. It was T2- and FLAIR- hyperintense and its localisation near the ventricle favoured TDL over GT (Fig. 1b,c). MRI of the atypical lesion fulfilled the reported characteristics of TDL [15–18]. However, MRI signs of TDL and GT can overlap easily [15, 17, 18]. Brain CT examination may be helpful in differentiation between the TDL and malignant GT [26] but we did not perform it in the early stage. Later, growth activity of the atypical lesion in the frontal lobe, good response to steroids, and absence of clinical signs suggesting tumour [15–18] led to treatment escalation. The effect of natalizumab and fingolimod to the lesion growth was evident and its MRI characteristics evoked suspicion of PML [11–13]. Although CSF examination of DNA JCV is a reliable test of PML, there were also reported cases of PML in JCV-PCR CSF negative patients in early PML as well as during Immune Reconstitution Inflammatory Syndrome (IRIS) [27].

In our patient, minimal mass effect and absence of post-contrast enhancement supported PML diagnosis [11–13]. Double Inversion Recovery (DIR) (Fig. 4c) confirmed cortical involvement but did not add new information. Cortical lesions are typical for advanced forms of MS but they can be found in PML as well as GT [11–13, 17–20]. Diffusion Weighted Imaging (DWI) hyperintensities (Fig. 3d) favoured PML [11–13]. ^1^H–MRS revealed decreased creatine to choline ratio in several areas of the lesion. We did not find changes of glutamate and glutamine peaks, as was reported by Yamashita [14]. ^1^H–MRS is able to identify brain tumours or demyelination [11, 28–31] although several authors recommend caution when interpreting ^1^H–MRS measured concentrations of metabolites in isolation [11, 31]. As our lesion was not well defined its precise place was estimated. Its localisation and size was finally revealed by ^11^C MET PET. Increased uptake of ^11^C–MET, i.e. high proliferation index of ^11^C–MET indicating malignancy [32], was found in the right cortical frontal area. Slightly increased uptake of ^11^C–MET was also found in left frontal and occipital cortex.

Cortical clinical symptoms, including disorientation, confusion, epileptic seizures, behavioural changes and headaches appeared in our patient in terminal stages of GT progression. They can also be found in patients with PML [8–11]. Moreover, symptomatic seizures correlate with cortical demyelination in advanced forms of MS [33], and could be associated with TDL [15, 16].

Basic CSF examination in our patient showed normal proteinorhachia and cell count, but increased number of oligoclonal bands type 2 (14 bands) and IgG index 1.44. These findings suggest active demyelinating processes associated with MS [15, 34] or PML-IRIS [10, 11]. This reaction is unusual in GT, where increased lactate in CSF and hyperproteinorrhachia would be expected [6]. However, we did not prove it.

Retrospective analysis of the blood cellular immunity over the years showed chronic increase of CD4+ (50–66%) and mild deficit of CD8 (11–12% with higher CD4/8 index (4.5–5.0) at the beginning of her disease (2007–2009). During interferon beta I treatment, CD4+ (55%) and CD8 (10%) remained unchanged, while CD16 + CD56 Natural Killers (NK) (4%, 66 abs) fell, and CD19 rose (31%, 513 abs). Natalizumab chronically decreased CD8 subpopulations of T lymphocytes. Fingolimod reduced CD4+ but significantly increased % of NK (49%). Humoral immunity remained normal and unchanged during all that time. Deficit of NK cells in our patient could decreased resistance against brain tumour growth, as NK cells can play an important role in anti-tumour immunity [35]. Moreover, it is known that in MS, autoimmune conditions are mediated mostly by CD4+ T-cells with a proinflammatory Th1 and Th17 phenotype, causing inflammation and demyelinating lesions in the CNS [36]. The dominancy of glial tumours in MS leads to a hypothesis that chronic hyperactivation of glial cells via Th1/Th17 pathways could cause their neoplastic transformation in demyelinated lesions [34]. On the other hand, if MS-associated proinflammatory Type17 T-cells mediate potent antitumour immunity [34], suppression and sequestration of those cells could potentially cause its breakdown. We did not test our patient’s Th17 lymphocytes, the mechanism could only be hypothesised. During natalizumab and fingolimod treatment we found lymphocytes decreased in periphery but we are not able to describe changes in the brain of our patient. This selective deficiency could have been involved in inefficient antitumour immune surveillance and tumour progression [37, 38].

Treatment by IMT could potentially trigger a variety of immunologic abnormalities that are typical for patients with malignant brain tumours [38, 39]. IMT may targeted many factors such as impaired responsiveness of peripheral blood lymphocytes to mitogens [39], failure of the T cells mediating adaptive immune responses within the local tumour micro-environment [40], and induction of regulatory T cells [41]. Development of GT can potentially be influenced by immunosuppressive cytokines (such as IL-10, TGF-β, and prostaglandin E2), and by down modulation of co-stimulatory molecules by antigen presenting cells (APCs) resulting in loss of T cell effector function [41]. Long-term monitoring of these markers in patients treated by highly effective IMT could be beneficial.

In tumour vessels, aggressive endothelial proliferation [42, 43], increases CD34+, a marker of endothelial progenitor cells [42]. Although natalizumab treatment results in an increase in CD34+ progenitor cells in both the bone marrow and the blood [44], it is not clear whether it can enhance tumour angiogenesis in a brain tumour. Moreover, increased angiogenesis was also described in PML lesions [11].

Indeed, the CNS biopsy remains the most useful method for defining the histological type of an atypical brain lesion susceptive of tumour. In our patient, Vimentin’s over-expression in cancer correlates well with increased tumour growth, invasion and poor prognosis [45]. OLIG2 is highly expressed in all diffuse gliomas [46] and was found expressed in our patient’s anaplastic astrocytoma samples. Tumourous cells were Nogo positive, Nogo-A exerts a growth inhibitory function leading to restricted axonal regeneration [47]. In our case Ki-67 was ≤5% but >2%. Worsened survival has been associated with Ki-67 > 2% [48]. Mutation of IFH1 and positive p53 in our patient suggests secondary nature of the astrocytoma [49].

In our patient we did not prove the histopathological triad of PML (multifocal demyelination, hyperchromatic, enlarged oligodendroglial nuclei, and enlarged bizarre astrocytes with lobulated hyperchromatic nuclei) [11, 27], which would have been a rather convincing evidence of the disorder. In Table 1, we summarise the differential diagnoses of tumefactive demyelinated lesions, malignant glial tumours, PML and PML associated with IRIS. We hypothesise that IMT may have transformed the glial cells into malignant GT. However, in a descriptive study among 22,563 French MS patients, including patients receiving IMT, brain tumours were not detected in total of 253 patients (1.2%) with a history of cancer [45]. In the SENTINEL trial of natalizumab in combination with interferon beta-1a, the incidence rate of cancer in the combination group (*n* = 589) was 1% compared to 2% in the interferon beta-1a alone group (*n* = 582) [50]. In interim analysis of TOP study, including 4821 patients from 16 countries, the incidence of malignancies was 0.5%. There were 24 patients with 12 types of malignancies. Glioblastoma was detected in 1 patient, while breast cancer was the most common, affecting seven patients (all female) [51].

**Table 1:** Differential diagnoses of tumefactive demyelinated lesions, malignant glial tumours, progressive multifocal leukoencephalopathy and progressive multifocal leukoencephalopathy associated with immune reconstitution syndrome

	TDL	malignant GT	PML	PML-IRIS	References
Clinical sy	motor, cognitive (aphasia, apraxia, agnosia, Gerstman, coma) sensitive, cerebellar, brain stem. Visual field defects, Epileptic seizures	epileptic seizures, cognitive and memory deficit, motor, sensitive.	altered mental status (aphasia), motor, limb and gait ataxia, visual symptoms: homonymous hemianopia, cortical blindness, diplopia. Epileptic seizures. No optic nerve and spinal cord involvement.	altered mental status (aphasia), motor, limb and gait ataxia, visual symptoms: hemianopia, cortical blindness, diplopia. Epileptic seizures. No optic nerve and spinal cord involvement	[8–14], [52]
MRI T2w/ FLAIR	Unilateral or bilateral. Frontal, parietal. Periventricular, juxta cortical. 2 cm, large lesion with little mass effect and edema Variable rate of hyper-intensity. Central dilated vessel. Irregular border. Supra-tentorial WM, partially GM involvement.	Unilateral. Supra-tentorial. Hyper or hypo intense. Irregular border. Central necrosis. Intensive vascular edema and mass effect	Bilateral. Supra-Intra-tentorial. Cortex, deep gray matter. (Frontal, parietal, occipital. Subcortical location, U-fibers, cortex, basal ganglia). 3 cm. Early new lesions. Hyper-intense. Ill-defined to WM, sharp to GM. No mass effect. Punctuate perilesional lesions.	Bilateral, spreading. Cortex and subcortical WM. Edema	[11–18], [16, 26, 54]
MRI T1w	Hypo-intense. CT- hypo-intensity	Hypo-intense. CT-hypo-intensity	Hypo-intense. CT-hypo intensity	Hypo-intense with hyper- intense rim	[11–18], [26, 54]
MRI Gd +	incomplete rim enhancement	complete rim enhancement	negative or variable, punctuate and rim like	positive or variable, punctate and rim like	[11–18], [26, 54]
MRI - PWI, DWI	decreased PWI in lesions, increased DWI in active demyelination	increased PWI, increased DWI in central necrosis	DWI always hyper-intense, with peripheral rim.	+/− restricted DWI	[9–18, 26]
1H- MRS	increased Cho, lipids, lactate, mildly decreased NAA and NAA/Cr (differ from malignant GT) elevation of the β,γ-Glx peaks	increased Cho and Cho/Cr, lipids, lactate, decreased NAA, lack of β,γ-Glx elevation	A decrease of NAA/Cr ratio, NAA and Cr. An increase of Lac/Cr, Cho/Cr, Cho, lipids/Cr, mIns, Lac, Lip.	increased Cho, decreased NAA, the presence of Lac/lipids at 1.3 ppm, and the presence of mIns, Higher Cho/Cr, mIns/Cr, Lip1/Cr, and Lip2/Cr in PML-IRIS than PML . Lower NAA/Cr than PML.	[11], [13, 14], [28–31]
CSF native	normal or mild increased proteinorhachia and white blood count. Positive oligoclonal bands, elevated IgG index	GFAP+ cells	mildly increased cellularity, normal proteinorhachia	mild to moderate increase of lymphocytes and protein levels	[10, 11], [14, 15]
CSF JCV DNA	negative, infrequently low positivity (less than 25)	negative? unknown	JCV-specific IgG, DNA copies, infrequently negative	JCV-specific IgG, DNA copies. Sometimes negative	[12–18, 31]
Response to corticoids	very good	only partial	none	good	[2, 9–11], [15, 27]
Histology	Hyper-cellularity, myelin protein-laden macrophages, variable lymphocytic inflammation, reactive gliosis and relative axonal preservation.	moderate cellularity, bizarre cells with hyperchromatic nuclei, moderate pleomorphic, gemistocytes, perivascular lymphocytes, rare areas of necrosis, neo-vascularization GFAP + cells: immature, reactive, and neoplastic astrocytes and ependymal cells	swelling of oligodendrocyte and multi-lobular astrocytes, basophilic nuclei, eosinophilic inclusion bodies, 2/3 of number of T cells in PML-IRIS. Positive DNA JCV staining in all types of cells. Active gene copies in high numbers of virally infected cells, as well as a low inflam- matory infiltrate.	Hyper-cellularity, CD8+ positive T cells dominate - their number the same as in active MS lesions, fewer CD4+ and CD20+ T cells in perivascular cuffs. High plasma and B cells in PML-IRIS-lesions. No or low number JCV-infected cells.	[2, 10], [15, 17–20, 49], [41–43, 46–48], [54, 55]
Outcome	as typical MS	progressive worsening	progressive worsening	worsening, regression possible	[6, 10–20, 49], [24, 25], [47, 48], [51]
Immunological markers in peripheral blood	upregulation of transcription factors of Th1 (pSTAT1 and T-bet) and Th17 (pSTAT3) in circulating CD4+, CD8 + T-cells and monocytes. CD4+ T-cells with a proinflammatory Th1 and Th17 phenotype,	lower T-bet, pSTAT1, and pSTAT3 in CD4+, CD8 + T-cells, and monocytes. Lower CD4 Th1 and Th17. Increased IL-10, TGF-β, PGE2, down modulation of co-stimulation molecules by APCs. Tumor angiogenesis, expression of CD34+ progenitor cells. Deficit of NK cells	variable data: stable CD4+ and CD8+, non-significant decrease or increased T cells but unchanged CD4/CD8 ratio. Decrease expression of CD49d, CD29 (VLA-4), CD11a, CD62L, CXCR3 on T cells. Decreased expression of VLA4 on myeloid dendritic cells, decreased count of dendritic cells. Production of CD34+ cells, increase of memory B cells. Increased IL-10.	Increased IFN-γ, IL-12p70, IL-4, IL-10, IL-5, IL-13. CD4+,T1 + PSTAT3+, CD8+, PSTAT1+,T-bet + . Decreased CD4+, CD25+ FoxP3+	[36–48, 56] [54]

*TDL* tumefactive demyelinating lesion, *GT* glial tumour, *PML* progressive multifocal leukoencephalopathy, *PML-IRIS* PML -immune reconstitution inflammatory syndrome, *MRI T2w* T2 weighted imagines of magnetic resonance imagine, *MRI T1w* T1 weighted imagines, *DWI* diffusion weighted imagines, *PWI* perfusion weighted imagines, *GAD* gadolinium enhancement, *WM* white matter, *GM* gray matter, *CT* computer tomography, *1H–MRS* proton magnetic resonance spectroscopy, *Cho* cholin, *NAA* N-acetyl aspartate, *Cr* creatine, *Lac* lactate, *mIns* myoinositol, *βγGlx* βγ glutamate + glutamin, *Lip* lipids, *NK* natural killers, *VLA4* alfa integrin 4, *IL 4,10,12,13* interleukin 4,10,12,13, *CD4* T helper lymphocytes, *CD8* T suppressor lymphocytes, *CD11* alfa component of various integrins, *CD20* B-lymphocyte antigen, *CD28* proteins expressed on T cells that provide co-stimulatory signals required for T cell activation and survival, *CD34+* hematopoietic progenitor cell antigen, *CD49* an integrin alpha subunit, *CD62* a cell adhesion molecule found on lymphocytes, *CD25+ FoxP3+* regulatory T cells, *IgG* imunoglobulin G, *CXCR3* a chemokine receptor that is highly expressed on effector T cells and plays an important role in T cell trafficking and function, *JCV* John Cunningham virus, *TGF-β* transforming growth factor β,PGE2 = prostaglandin E2, *GFAP* glial fibrillar acidic protein, *STAT 1,3* Signal transducer and activator of transcription 1,3, *Th1* T1 lymphocytes, *Th17* T17 lymphocytes, *T-bet* transcription factor, *APC* antigen presenting cells, *CSF* cerebrospinal fluid, *DNA* deoxyribonucleic acid

Our patient was the carrier of human leukocyte antigens (HLA)-DRB 1*15 and DRB 1*11 alleles, and heterozygote of single nucleotide polymorphism rs3135388, which is typical for multiple sclerosis [52]. Although expression of HLA antigens is important for the immune response against infectious agents and malignant cells, there is an information gap about the link between HLA antigens and brain glial tumours. However, one prospective study, focused on HLA typing of German Caucasian patients, found that HLA-DRB1*15 in combination with HLA-DRB1*11 was associated with higher (a 13.4-fold increased) risk of glioma than was found for other HLA alone or in other combination [53]. It might explain the occurrence of astrocytoma Grade III in our patient.

We suppose that the anaplastic astrocytoma in our patient, developed after the diagnosis of MS, could have arisen in demyelinative plaques with reactive gliosis and could have evolved from a low grade glioma to anaplastic astrocytoma over time. The role of the immunomodulatory treatment as well as other immunological factors in malignant transformation of the tumour can only be hypothesised. The association between gliomas and MS is uncommon but it must be kept in mind when an atypical tumefactive lesion develops in a patient with MS. In our case, it is the first time when malignant glioma was misdiagnosed as PML.

## Abbreviations

^11^C–MET PET: ^11^methyl-methionine positron emission tomography
^1^H MRS: ^1^H–proton MR spectroscopy
APC: Antigen Presenting Cell
CNS: Central Nervous System
CSF: Cerebrospinal Fluid
CT: Computer Tomography
DNA: Deoxyribonucleotid Acid
EDSS: Expanded Disability Status Scale
GFAP: Glial Fibrillary Acidic Protein
GT: Glial Tumour
HE: Hematoxylin and Eosin
HLA: Human Leukocyte Antigen
IDH: Isocitrate Dehydrogenase1
IL-10: Interleukin-10
IMT: Immuno-modulatory Treatment
IRIS -: Immune Reconstitution Inflammatory Syndrome
JCV: John Cunningham virus
Ki-67: a nuclear protein that is associated with cellular proliferation
MRI -: Magnetic Resonance Imaging
MS: Multiple Sclerosis
Nogo-A: a high molecular weight transmembrane protein expressed by oligodendrocytes in white matter of the CNS
Olig2: Oligodendrocyte transcription factor
p53: cellular tumour antigen
PCR: Polymerase Chain Reaction
PML: Progressive Multifocal Leukoencephalopathy
SV40: Simian Virus 40
TDL: Tumefactive Demyelinating Lesion
TGF-β: Transforming Growth Factor-β
VEP: Visual Evoked Potentials
